# Implementation of the community health system innovation project in three low- and middle-income countries: COHESION-I study protocol

**DOI:** 10.1136/bmjopen-2025-109433

**Published:** 2025-12-31

**Authors:** Maria Lazo-Porras, Antonio Bernabe-Ortiz, Albertino Damasceno, Sanjib Kumar Sharma, Devarsetty Praveen, Nikol Mayo-Puchoc, Nathaly Aya Pastrana, Mariella Bazán Maccera, Anibal Chauque, Lucero Cahuana-Hurtado, Maria Kathia Cardenas, Ujwal Gautam, Vijay Kumar Khanal, Neusa Jessen, Nelson Mugabe, Rocio Pereyra, Maria Amalia Pesantes, Suman Bahadur Singh, J Jaime Miranda, David Beran

**Affiliations:** 1 CRONICAS Center of Excellence in Chronic Disease, Universidad Peruana Cayetano Heredia, Lima, Peru; 2 Faculty of Medicine, University of Eduardo Mondlane, Maputo, Mozambique; 3 Research Unit, Department of Medicine, Maputo Central Hospital, Maputo, Mozambique; 4 BP Koirala Institute of Health Science, Dharan, Nepal; 5 George Institute for Global Health India, New Delhi, India; 6 The George Institute for Global Health, University of New South Wales, Sydney, New South Wales, Australia; 7 Prasanna School of Public Health, Manipal Academy of Higher Education, Manipal, Karnataka, India; 8 IMEK Centro de Investigación en Mercadeo & Desarrollo, Santiago de Cali, Valle del Cauca, Colombia; 9 Faculty of Public Health, Universidad Peruana Cayetano Heredia, Lima, Peru; 10 Dickinson College, Carlisle, Pennsylvania, USA; 11 Faculty of Medicine and Health, School of Public Health, The University of Sydney, Sydney, New South Wales, Australia; 12 Division of Tropical and Humanitarian Medicine, Faculty of Medicina, University of Geneva and Geneva University Hospitals, Geneva, Switzerland

**Keywords:** Community Participation, Diabetes Mellitus, Type 2, Hypertension, Neglected Diseases, Patient Satisfaction, PUBLIC HEALTH

## Abstract

**Background:**

The COmmunity HEalth System InnovatiON (COHESION) project (2016–2019) was a 4-year collaboration between research teams from Mozambique, Nepal, Peru and Switzerland. It conducted formative health system research using tracer chronic conditions, non-communicable diseases (diabetes and hypertension) and one neglected tropical disease per country (schistosomiasis in Mozambique, leprosy in Nepal and neurocysticercosis in Peru).

Findings guided the co-creation of interventions to improve diagnosis and management through a participatory approach with communities, primary healthcare workers and regional health authorities.

As a continuation of this effort, the research team initiated the COHESION Implementation project (COHESION-I) with two objectives: (1) implement and evaluate the context-specific co-created interventions in Mozambique, Nepal and Peru (Component 1) and (2) adapt the COHESION approach to India, a country that did not benefit from a formative phase previously (Component 2). This protocol manuscript focuses on Component 1.

**Methods and analysis:**

A mixed-methods, pre–post quasi-experimental design will be used, including quantitative, qualitative, economic and process evaluations. Each country will have three arms: (1) co-created and co-designed interventions; (2) only co-designed intervention and (3) the usual care arm. Data will be collected longitudinally over 18 months to assess the effect of the interventions. The main outcomes include patient satisfaction (Patient Satisfaction Questionnaire Short Form), health system responsiveness (WHO responsiveness domains) and quality of life (EuroQol 5 dimensions 5 levels). The qualitative evaluation will explore how satisfaction is perceived among service users with chronic conditions and healthcare workers. Other outcomes per type of evaluation will be considered such as perceived value of health services, cost estimation and acceptability of the intervention components, among others.

**Ethics and dissemination:**

Approvals were obtained from Ethics Committees of Universidad Peruana Cayetano Heredia (Peru), Universidade Eduardo Mondale (Mozambique) and Nepal Health Research Council (Nepal). Results will be disseminated through peer-reviewed publications and scientific conferences.

**Trial registration number:**

NCT06989502.

**STRENGTHS AND LIMITATIONS OF THIS STUDY**

- This protocol provides an approach for designing complex context-adapted interventions that engage the voices of diverse stakeholders in three low- and middle-income countries.
- Our research proposes a comprehensive evaluation framework integrating quantitative, qualitative, economic and process evaluation to assess intervention outcomes.
- The initial survey will inform about the individuals with the conditions studied, and the population satisfaction and responsiveness to adjust sample sizes per arm.
- The evaluation will focus on low-resources primary healthcare centres, where system constraints may limit effectiveness or it may have substantial impact due to these populations rarely receiving health interventions.

## Background

 The management of chronic diseases poses a problem to many health systems in low- and middle-income countries (LMIC) as health systems in these countries are not organised to provide long-term care.^1^,^3^ Managing chronic diseases requires a comprehensive health system, with regular availability of medicines, laboratory facilities, data collection tools, trained healthcare staff and educated and empowered service users.^1 4^

To address this, the Chronic Care Model was adapted to LMICs under the name of the Innovative Care for Chronic Conditions Framework (ICCCF).^4 5^ The ICCCF calls for a ‘paradigm shift’ to ensure that health systems are capable of organising care for chronic conditions and includes changes at policy level, health system and in communities. Sadly, in LMICs, the lowest utilisation of public health services is by the poor, those who need it the most, as these services are often not available or sensitive to their needs.^6 7^ Weaknesses in health systems and their inability to address the health needs of vulnerable populations, particularly at the primary healthcare (PHC) level, are well known barriers to achieving people-centred care.^8^ In order to reach this goal, it is essential to improve healthcare quality and strengthen the health system to ensure it is more responsive to people’s needs. Also, it has been shown that focusing on the community as recipients of care and the issues that affect healthcare workers, as providers of this care, can have better yields.^2^

The management of chronic diseases improves if the patient is satisfied with the health services.^9^ However, the evaluation of satisfaction is not always aligned with the performance of the health system and the use of responsiveness was proposed. Some challenges to measure satisfaction are that people living in vulnerable conditions may not always feel comfortable expressing their opinions through surveys, while interviews appear as a suitable strategy for people to share their views.^10^ These qualitative approaches allow us to elucidate service users’ expectations from the local health services, which are also crucial to understand whether a health system is considered responsive.^11^ In order to understand the underlying issues related to the provision of quality services, WHO proposed the health systems responsiveness framework, which is useful to focus on the interaction between service users and healthcare workers, going beyond specific diseases and leading to a true strengthening of the health system. For these reasons, patient satisfaction and responsiveness are good proxies of health system performance and useful to disentangle the complexity of the health system and evaluate its performance from a people-centred care perspective.

A recent cross-sectional study in middle-income countries on healthcare quality and health system responsiveness concluded that rigorous evaluations of interventions to improve quality of care and responsiveness are urgently needed, given the importance of both elements for achieving effective universal health coverage (UHC).^3^ Furthermore, the WHO states that there is a positive association between health outcomes and responsiveness,^11^ and evidence suggests that demand-side barriers may be as important as supply factors in deterring service users from obtaining chronic care.^12^ Although many state that co-designed interventions are more successful in achieving satisfaction and better health outcomes, this has not been truly tested.^13^ Therefore, new approaches are needed to generate evidence from health systems research in LMIC about the practices to improve patient satisfaction and responsiveness derived from the expectations and demands of those who provide care (healthcare workers) and those who use the health system (service users).

### Objectives

To implement and evaluate, at the PHC level, the co-created/co-designed interventions in the three countries (Mozambique, Nepal and Peru), focusing on non-communicable diseases (NCDs) (ie, diabetes, hypertension) and neglected tropical diseases (NTDs) (ie, schistosomiasis in Mozambique, leprosy in Nepal and neurocysticercosis (NCC) in Peru) to improve patient satisfaction and health system responsiveness. The present protocol has a comprehensive approach to evaluate their results and, therefore, the general objective is subdivided into four subcomponents of evaluation:

- *Quantitative evaluation*: To evaluate changes in the level of patient satisfaction and health system responsiveness among PHC users living with a chronic condition. For this, a pre–post strategy approach will be used with two assessments before the implementation of the intervention, one assessment during the intervention and two assessments after the intervention, with a 3-month time gap between assessments.
- *Qualitative evaluation*: To explore the differences that exist between the perceptions of PHC users living with a chronic condition about the local public health services (*through time*)*,* and the perceived roles and responsibilities healthcare workers feel they have toward PHC users (*over time*)*,* and how these differences change (increase or decrease) as the interventions are implemented.
- *Economic evaluation*: To assess the costs and cost-effectiveness of co-designed interventions compared with usual care to improve the health system responsiveness and satisfaction of people with chronic conditions at the PHC level.
- *Process evaluation*: To explore contextual factors that may impact the implementation of COmmunity HEalth System InnovatiON Implementation (COHESION-I), describe the implementation process of the project and identify the mechanisms through which the implemented intervention has an impact (or not) in the arms that receive the intervention.

### Hypotheses

The primary hypothesis of the COHESION-I study is that patient satisfaction and health system responsiveness will be higher in the intervention arms (co-creation/co-design and only co-design interventions) compared with the usual care arm in each country. We also hypothesise that, between active interventions, greater satisfaction and responsiveness will be observed among those in the co-created/co-design intervention compared with only co-design intervention.

## Methods and analysis

### Context

The COHESION project was a 3-year project that started in 2016 as a collaboration between teams from Mozambique, Nepal, Peru and Switzerland funded by the Swiss National Science Foundation and the Swiss Agency for Development and Cooperation as part of the Research for Development programme (r4d Programme). The COHESION project conducted formative research at policy, health system and community levels using NCDs and NTDs as tracer conditions. The NCDs were diabetes and hypertension (HTP), and in the case of NTDs, it included schistosomiasis in Mozambique, leprosy in Nepal and epilepsy resulting from NCC in Peru.

The results from this formative research were used to identify adequate interventions through a participatory approach with communities, healthcare workers from PHC and regional health authorities (co-creation). Meetings with different stakeholders took place in 2017 and 2018 to propose context-relevant interventions oriented to address the challenges of providing care for people affected by diabetes, HTP and the selected NTDs (schistosomiasis, leprosy and epilepsy due to sequelae of NCC). During the meetings, participants provided feedback regarding problems and potential solutions for chronic care and health services in general and proposed possible areas of intervention.^14^ According to the needs in each country, other activities were used to strengthen the health system response and achieve a more effective communication. The results of the co-creation are available in online supplemental table 1.

### COHESION-I

The COHESION-I project is the continuation of the COHESION project. It will be implemented between 2022 and 2026 (4 years) and has two components. Component 1 involves Peru, Mozambique and Nepal and has two parts: the first one is the co-design process, and the second one is the implementation and evaluation of the interventions. Component 2 is the optimisation of the process of formative research, co-creation and co-design in a new country, India. This protocol describes the work to implement Component 1.

The project defines co-design as the process of involving different stakeholders in designing or refining interventions for a prespecified problem, using participatory approaches to generate creative strategies.^15^ Co-design in COHESION-I seeks to refine the interventions previously co-created (online supplemental table 1). The research team is currently co-designing communication materials, training formats for healthcare workers and coordinating health facility improvements. This process ensures the proposed interventions reflect people’s values, perceptions and expectations around health service delivery.^16^ The three countries initiated the fieldwork activities of the co-design process in May 2023, and it will end in early 2026. This manuscript protocol focuses on the implementation and evaluation of the co-designed interventions.

### Setting

The implementation of interventions in COHESION-I will be conducted in Mozambique, Nepal and Peru, countries with different characteristics and health systems responses to chronic conditions as it has been described in detail elsewhere.^17^ The sites were selected during the first phase of the project to capture diversity in geography, socioeconomic conditions and health systems structures in three different continents. Moreover, selected sites prioritised locations where the co-creation approach had been applied, and they included urban and rural sites.^14^ Each country will have two intervention arms and one control arm.

### Health system in the selected countries

This section provides a brief description of the health systems in each of the countries to understand better the contexts where the COHESION-I interventions will be implemented and evaluated.

#### Nepal

Every citizen in Nepal is entitled to free basic health services as health is considered a fundamental human right in the constitution. The health system in Nepal is decentralised and structured in three tiers: local/municipal level, provincial level and federal level. Provision of primary health services is the responsibility of local government. Provincial government is responsible for delivery of basic health services through provincial hospitals and coordinating with local and federal levels. The federal government is responsible for policy formulation, budget allocation and delivery of specialised healthcare through national hospitals. Primary health services at local level are delivered through PHC centres, Health posts, Basic Healthcare Service Centres, Urban Health Centres and primary level hospitals.^18^ Besides this, the primary level often extends its services to communities through PHC outreach clinics, female community health volunteers and immunisation clinics.^18^ In order to ensure UHC, the Government of Nepal has employed Social Health Insurance Schemes that offer financial protection to service recipients in PHC centres and every tier of hospitals.^19^

#### Mozambique

The Mozambican health system created after the independence of the country in 1975 is based on the Alma Ata conference, giving the higher priority to the PHC system.^20^ The health sytem in Mozambique is structured in primary, secondary and tertiary care. The PHC centres are public; although the network has some geographic gaps, the system was designed predominantly to take care of infectious diseases that were predominant.^21^ This means that NCDs are still a challenge in terms of training of the health team, availability of diagnostic basic instruments and availability of drugs. The major priorities for the system are HIV, tuberculosis and malaria programmes that are almost totally financed by the external help. All the PHC activity is almost free for all. The private health system is limited to the big cities and is very small and non-significant. Most of the population is not covered by a system of health insurance.^21 22^

#### Peru

The Peruvian health system is a fragmented, labour-based health system, with stewardship responsibilities falling under the Ministry of Health. The Ministry of Health provides care for people with low income (70% of the population), while Social Security offers services to formal workers and their families (20% of the population). There are also health services for the armed forces and also a private sector.^23^ The health system is organised in primary, secondary and tertiary healthcare levels and specialised institutes. Each of the 28 health regions in the country is responsible for the resource allocation to the three levels.^24^

### Study design

The COHESION-I project will implement a co-created/co-designed intervention and concurrently evaluate the intervention using a mixed-methods approach that includes a pre–post quasi-experimental study to assess the effect of the co-designed and co-created interventions, the co-design intervention on the primary and secondary outcomes, compared with the usual care. The project will have four types of evaluations that have a different approach and design. These are the following:

#### Quantitative evaluation

The quantitative evaluation will set two different approaches. First, an initial survey will be conducted to have appropriate estimates of the PHC users and validate tools to be used in the intervention assessment. Second, a quasi-experimental approach will be used to assess the effect of the co-designed and co-created interventions on the primary and secondary outcomes compared with the usual care in an 18-month period.

#### Qualitative evaluation

The qualitative evaluation will include two studies: a qualitative longitudinal study with local PHC users and a qualitative study with healthcare workers. The qualitative evaluation will be guided by the ‘lay evaluation of healthcare’ theoretical framework proposed by Calnan,^25^ value theory^26^,^29^ and the WHO responsiveness framework.^30^

The longitudinal qualitative study aims to explore the experiences of adult women and men living with diabetes, hypertension or the specific neglected tropical disease and how they unfold *through time*^31^. The data will be collected at different moments (initial survey, moments 3 and 5) with the same participants through in-depth and semi-structured interviews. Photo-elicitation will be used, if participants accept, to evoke conversations during the interviews.

The qualitative study with healthcare workers will examine *over time* the perceived roles and responsibilities of healthcare workers in providing care for PHC users with chronic conditions. The interviews will be conducted at three different moments, before, during and after the intervention implementation.

#### Economic evaluation

An economic evaluation will be conducted, comparing two arms: care-as-usual (control) and the implementation of the co-design interventions. The evaluation will be conducted in three distinct phases: (1) implementation only, (2) co-design and implementation, and (3) co-creation, co-design and implementation. A cost-effectiveness and cost–utility analysis will be conducted. The study will adopt a dual perspective, examining both the healthcare sector (ie, the Ministry of Health of each country) and the societal (ie, households) perspectives. The time horizon over which costs and consequences will be evaluated is 3 years, and the discount rate will be 10% according to national rates. The relatively short time horizon and the discount rate will be considered as parameters in a sensitivity analysis.

#### Process evaluation

The evaluation is based on the Medical Research Council (MRC) as the core framework^32^ and is complemented with the normalisation process theory (NPT).^33^ The MRC framework explores the contextual factors that interact and influence during the implementation of the intervention.^34^ We will focus on (1) monitoring the intervention delivery and (2) the reception of the intervention, in terms of the following subcomponents: reach, dose (delivered and received), fidelity and acceptability.^34^ NPT is a theory that explains the social processes when implementing complex behavioural interventions in the healthcare practice.^33^ The four constructs (coherence, cognitive participation, collective action and reflexive monitoring) will be assessed in the people who have received some components of the intervention and can be examined using the Normalization Process Theory Questionnaire (NoMAD tool).^35^ The process evaluation will encompass a mixed-method approach using surveys, observations, checklists and interviews with different actors.

**Figure 1:**
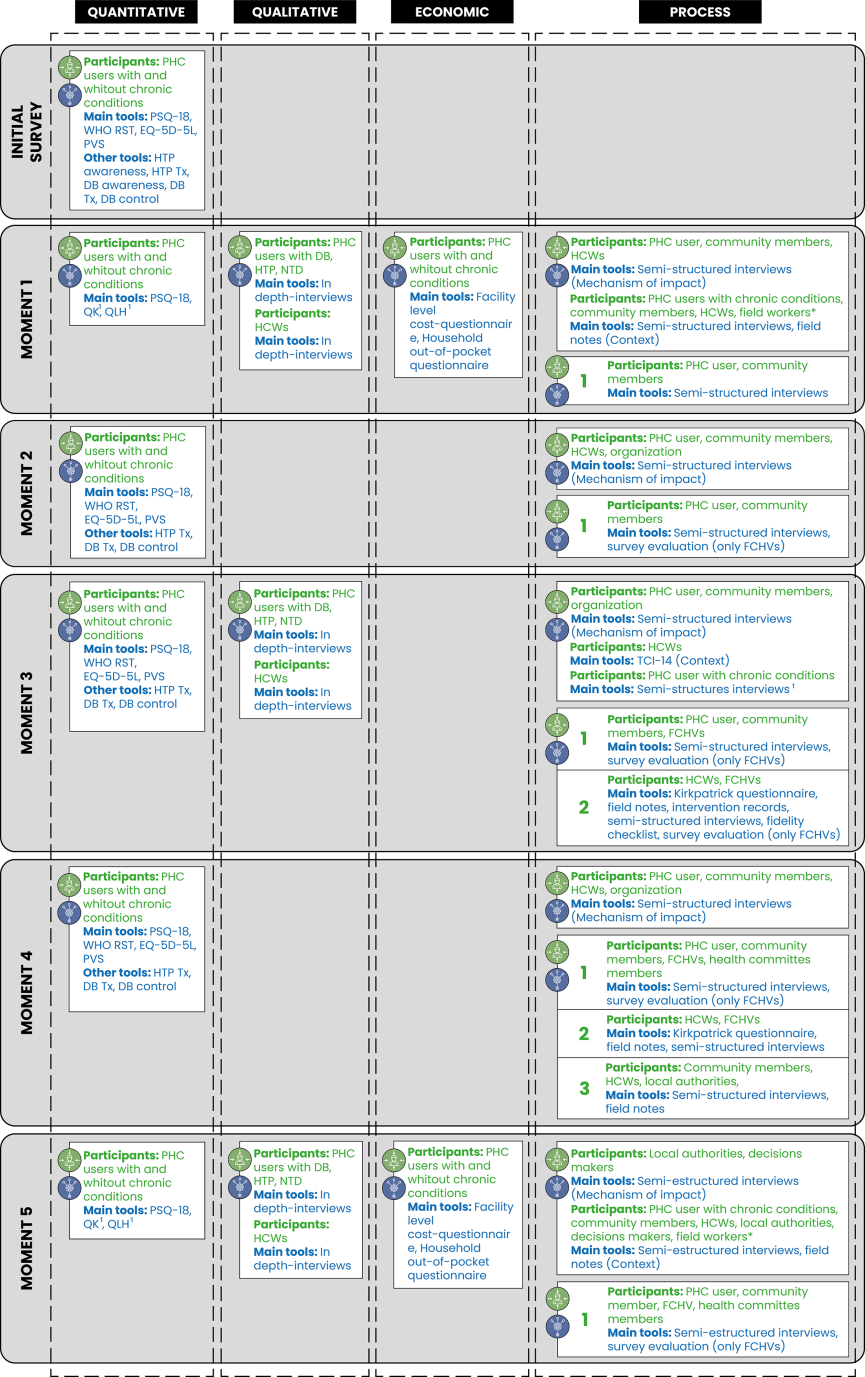
Graphical representation of the implementation of COmmunity HEalth System InnovatiON Implementation (COHESION-I) project. ^1^The implementation of the communication strategy is integrated within the quantitative and process evaluation. The process evaluation has three types of evaluation: Mechanism of impact, Context and Implementation outcomes (1 = community, 2 = healthcare workers and 3 = health facility). *Field workers will participate at moment 5 by making field notes on the project context. WHO RST, World Health Organization Responsiveness; EQ-5D-5L, EuroQol 5 dimensions 5 levels; FCHV, female community health volunteers; HCW, healthcare worker; NTD, neglected tropical disease; PHC, primary healthcare; PSQ-18, Patient Satisfaction Questionnaire Short Form; PVS, People’s Voice Survey; HTP, hypertension; DB, diabetes; Tx, access to treatment and medication; QK, Questionnaire of knowledge; QLH, Questionnaire on lifestyle habits.

Figure 1 shows the different evaluations, moments and tools to be used.

### Participants and selection criteria

Table 1 details the general characteristics of participants across the three countries and the criteria used for the selection of participants. The COHESION-I project involves seven types of key actors at the macro, meso and micro levels of the health system that will participate in each type of evaluation. These participants will be recruited in all arms.

**Table 1:** Participants and inclusion criteria

Component	Level	Type of participant	Inclusion criteria
Quantitative evaluation	Micro	PHC users	18 years of age or older. Live in one of the selected rural, peri-urban or urban sites in the three countries.
		PHC users with chronic conditions	18 years of age or older. Live in one of the selected rural or suburban sites in the three countries. Have a diagnosis of diabetes or hypertension or selected NTD in each country. To complement the levels of low awareness, measurements will be taken in those 30 years of age or older (initial survey). The criteria are: having blood pressure (≥140 mm Hg systolic, or ≥90 mm Hg diastolic, average of two readings) and/or having a high risk of diabetes by FINDRISC and a fasting glucose test.
Qualitative evaluation	Micro	PHC users with chronic conditions	18 years of age or older. Live in one of the selected rural or suburban sites in the three countries. Have an established diagnosis of the disease for at least 2 years at the time of the first interview. Be health services users (female or male) with chronic conditions. Have used the local PHC services in the last 12 months.
		Healthcare workers*	Be a healthcare worker who provides the health services in the local PHC facilities in the three countries. Be involved in the non-communicable and/or neglected tropical diseases department (not exclusive).
Economic evaluation	Micro	PHC users with chronic conditions	Same inclusion criteria of the quantitative evaluation.
Process evaluation	Macro	Decision makers from national government*	Have a role in the national state of the three countries during the period of the interviews. Have a role in the non-communicable and/or neglected tropical diseases department (not exclusive).
	Meso	Decision makers from regional/state/local government*	Have a role in the regional state of the three countries during the period of the interviews.
	Micro	PHC users with chronic conditions	Be health service users with chronic conditions. Have used the health services in the previous 3 months.
		Not PHC users	18 years of age or older. Not be health service users or people who have not used PHC services for at least 12 months.
		Healthcare workers	Be a healthcare worker who provides the health services in the local PHC facilities in the three countries.
		Community healthcare workers†	Female community healthcare volunteers and members of the health committees. Have participated in the training of the intervention.
		Local authorities	Be a local authority in one of the selected rural or suburban sites in the three countries. Be a local authority during the period of the interviews and have knowledge about the situation of their community.

* Ideally, participants will be the same in the different measurements.

† Only for Nepal and Mozambique.

FINDRISC, The Finnish Diabetes Risk Score; NTD, neglected tropical disease; PHC, primary healthcare.

To complement the low awareness in Peru regarding blood pressure levels and diabetes diagnosis, the quantitative and economic evaluation will conduct a two-step diabetes screening. In order to conduct the diabetes screening, the Finish Diabetes Risk Score (FINDRISC) will be used and a fasting glucose in those identified at high risk in the countries will be measured.^36^ In the three countries, to recruit participants for the qualitative study, the research team will contact local leaders from sites and health promoters and, after explaining the aim of the COHESION-I project and the study, they will be asked for references of community members meeting the selection criteria for this study. Recruitment strategies will aim to have a similar number of women and men participating in the study.

### Intervention

The intervention in the three countries was decided using co-creation as it was described in previous studies^14 37^ and it will be delivered after approximately 6 months from the initial survey. The intervention will last 7 months.

During the co-creation process, different stakeholders participated in defining its components in the COHESION project. Each of these components was aimed at promoting change at the community level, among healthcare workers and within health facilities. Specific details of each component will be decided during the co-design process. However, some key aspects are already decided, and the Template for Intervention Description and Replication (TIDIeR) checklist^38^ is used to share this information (table 2).

**Table 2:** Intervention description using Template for Intervention Description and Replication (TIDIeR)

	Peru	Mozambique	Nepal
*Why and what*	During the formative studies and co-creation process, it was decided to include: **Radio programmes, spots or podcasts** to motivate service users to clarify concerns about their health condition and treatment. For healthcare workers, ***c*apacity building on management of diabetes, hypertension and NCC, as well as clear communication** will be included. The training will provide a set of skills that are lacking between healthcare workers. **A communication tool** will be developed to use during consultation to promote more interaction between healthcare workers and service users. The intervention will also include **a component related to decentralised decision-making to enhance PHC facilities**. This component will consist of a small grant for the community to decide how to spend the money to improve the equipment and/or infrastructure of the health facility.	Similar to Peru, it was decided to include **a communication strategy** as well as **capacity building for healthcare workers**. However, the communication strategy will use other channels such as videos through PHC TV. Likewise, an ‘**information booth’** will be established to use in the health facility to improve the communication between healthcare workers and user services. This booth enables healthcare workers to be better informed and offers clear information to service users. In addition, **group discussions will be performed between healthcare workers to express their challenges and opportunities during the consultation**, in order to have a better comprehension of each user, identify needs and issues more efficiently. Regarding the health facility, it will include a component related to the **improvement of the appointment system (similar to the one available for HIV) and the advocacy on the issue of access to medicines**. This component aims to improve the quality of basic needs in the health facility.	Similar to Peru, it was decided to include **a communication strategy,** as well as **capacity building for healthcare workers**. In addition, this component will include the **participation of female community health volunteers**. Also, a **flip chart and guidelines** for management of diabetes and hypertension will be created and also will be part of the training for healthcare workers to provide the guide and confidence to attend to the needs of patients in the consultation. **The health facility component will adopt a similar approach to that in Peru**. It will consist of **posters on diabetes and hypertension, supplemented by questions to have a better understanding of health conditions** and improve communication between health staff and service users.
Who provided	The radio programmes, spots or podcasts content will be created with input of people with different expertise such as NCDs, NTDs, local authorities, service users, healthcare workers and experts in behavioural sciences and communication. The training content for healthcare workers will be developed and delivered by experts in PHC and medical education as well as local trainers. The communication tool will be used by the healthcare workers, and they will be trained by the research team.	The communication component, as well as the training for healthcare workers, will follow a similar approach to Peru. The ‘information booth’ and group discussions will be implemented in the health facilities and will be conducted by healthcare workers. The appointment system and the medication equity promotion will be structured by the research team, healthcare workers of health facilities, experts in PHC and local authorities.	The communication component will be developed by a diverse group of experts, reflecting a similar approach to Peru and Mozambique. Similarly, the training structure for the healthcare workers component will be crafted by seasoned professionals in PHC and medical education, complemented by the cultural insights of local trainers. On the other hand, collaboration among healthcare workers, the research team, and community stakeholders will inform the development of the facility component, which entails implementing posters addressing clinical conditions.
How	The radio programmes, spots or podcasts will be delivered through the local community radio/loudspeaker or social media (WhatsApp or Facebook) to disseminate the information. The training for healthcare workers will be provided face-to-face with some additional resources and information delivered through videos and/or documents. The communication tool will be printed, and the cards will be available in each healthcare worker consultation of the health facilities. The facility grant will be provided by the research team according to a predefined budget.	The communication component will also be delivered through TV or social media (Facebook and WhatsApp) to disseminate the information. The training will be imparted principally face-to-face and complemented with media resources. The material for the ‘information booth’ will be printed and produced to be available at the health facilities. Similarly, material for the promotion of access to medication will be developed and made available at each centre.	The community component will be disseminated via radio or loudspeaker systems, or through social media platforms (Facebook) as observed in other countries. Healthcare worker training will be conducted in person and complemented with supplementary resources. Flip charts and guidelines will be developed and printed for availability to healthcare workers during consultations. Additionally, concerning the health facility component, the poster materials will be printed and strategically placed within the health facility to assist healthcare workers.
Where	The intervention occurred within the PHC level, most of the activities are in the health facility but some of them could be part of the home visits.		
When and how much	Many of these characteristics will be defined in the co-design process.		
Tailoring	The intervention is tailored to the needs and expectations of the local contexts as a result of the ongoing co-design process. However, due to the diversity of community members, the research team cannot ensure the co-designed tools will fit everybody.		

NCC, neurocysticercosis; NTD, neglected tropical disease; PHC, primary healthcare; TV, television.

### Outcome

The primary and the secondary outcomes for each component are detailed in table 3 and online supplemental tables 2, 3.

**Table 3:** Outcomes

Evaluation type	Outcome level	Representative outcomes	Measurement instruments/data sources	Collected from	Time
Quantitative evaluation	Primary outcomes	Patient satisfaction; health system responsiveness; People’s Voice Survey; quality of life	PSQ-18; WHO responsiveness tool; People’s Voice Survey; EQ-5D-5L	PHC users with chronic conditions (HTP, DB, NTD); PHC users	Initial survey; M2–M5
	Secondary outcomes	Access to care and treatment for HTP and DB; disease control (blood pressure, glycaemia); health literacy and behaviours (diet, service use)	Self-reported surveys; blood pressure and glucometer measurements	PHC users with chronic conditions (HTP, DB, NTD); PHC users	Initial survey; M2–M5
Qualitative evaluations	Primary outcomes	Perceived value of care; patient satisfaction; roles and responsibilities; perceived capacity	In-depth interviews; interviews	PHC users with chronic conditions (HTP, DB, NTD); HCWs	Initial survey or M1, M3, M5
Economic evaluation	Primary outcomes	Cost of services provided; out-of-pocket expenses; cost per QALY gained; cost per satisfied patient; cost per responsive patient	Facility-level cost questionnaire; household out-of-pocket questionnaire; costs calculated previously	PHC users with chronic conditions (HTP, DB, NTD); PHC users	M1, M5
Process evaluation	Context	*Barriers and facilitators:* environmental context during COHESION-I implementation; community and regional support; influences on intervention; team dynamics	Fields notes; meso-level and local leader questionnaires; Team Climate Inventory	Field workers; decision-makers; community members; PHC users with chronic conditions; non PHC users; HCWs	M1, M5
		*Health system-level indicators:* service delivery and workforce performance; access and affordability of care; patient empowerment; community engagement	Semi-structured interviews; service evaluation checklists; facility and stock records; brief provider and patient surveys	Community members; PHC users with chronic conditions; HCWs	M1 - M5
	Implementation outcomes	Online supplemental table 3*			
	Mechanism of impact	Coherence; cognitive participation; collective action; reflexive monitoring (NPT constructs)	Semi-structured interviews using NPT framework	PHC users, community members, HCWs; decision-makers; local authorities	M1 – M5 (variable, see online supplemental table 2)
Communication strategy†	Quantitative evaluation perspective	Comprehension of chronic disease concepts; risk awareness; preventive health behaviours	Questionnaire of knowledge; questionnaire on lifestyle habits	PHC users; PHC users with chronic conditions (HTP and DB)	M1, M5
	Process evaluation perspective	Exposure to themes of communication strategy; remembrance and comprehension of communication messages on chronic diseases	Semi-structured interviews	PHC users with chronic conditions (HTP and DB)	M3

* For the implementation outcome from the process evaluation, the online supplemental table 3 was developed.

† The evaluation of the communication strategy will be integrated into the quantitative and process evaluation.

COHESION-I, COmmunity HEalth System InnovatiON Implementation; DB, diabetes; EQ-5D-5L, EuroQol 5 dimensions 5 levels; HCWs, healthcare workers; HTP, hypertension; M, Moment; NPT, normalisation process theory; NTD, neglected tropical diseases; PHC user, user who received care in one of the primary healthcare facilities; PSQ-18, Patient Satisfaction Questionnaire Short Form; QALY, quality-adjusted life-years.

### Sampling methods

#### Sample size for the quantitative and economic evaluation

Few studies of health system responsiveness have been conducted in LMICs,^3 39 40^ none in Mozambique, Nepal or Peru. To guide our sample size calculation, we used the estimations of a Chinese study designed to improve patient satisfaction (PSQ-18) through a 2-week feedback intervention directed to physicians.^41^ This study did not consider the repeated-measure structure of the research but was the only one available at the time of preparing this article. Therefore, we will revisit estimations after the initial survey to validate our assumptions. The means (SD) of PSQ-18 at baseline were 3.38 (0.48) and 3.37 (0.52) for the control and intervention groups, respectively. At the follow-up, these were 3.44 (0.48) and 3.69 (0.55), respectively. Using this information, we estimated the sample size required for a repeated-measure analysis of interaction between groups and measures. Estimations were conducted in STATA using the ‘power repeated’ command. First, we defined a matrix of effect and covariances according to the aforementioned. Then, a within-individual correlation of 0.3, a type I error of 0.05 and a power of 90% were assumed. Considering these parameters, it was estimated that the project would need at least 121 participants per arm (242 per arm), considering a 10% loss to follow-up. Given the health system is more commonly used by women, we stratified our sample by sex to include 60% females and 40% males. Given the community size, population structure and disease burden (for those with NCDs or NTDs), we anticipate having enough cases to observe changes between interventions and usual care arms. In addition, a sample of 100 PHC users without chronic conditions will also be evaluated in each PHC centre. This sample is considered because the project aims to evaluate the effect on the primary outcomes not only in those PHC users with chronic conditions, but it will also measure its effects in PHC users with other conditions in order to evaluate changes in the health system beyond the management of chronic conditions.

#### Sample size for the qualitative and process evaluation

##### PHC users (n=8 interviewees/arm): qualitative evaluation

In each of the three arms, eight people will be interviewed. A total of 24 interviews per country will be conducted per data collection moment. Out of the 24 participants, 12 should be between 30 and 45 years old, and the rest above 45 years old. In each arm, we will aim to have at least two males and two females with HTP, two females with the selected NTD, and two males and two females with diabetes. The same participants will be interviewed at the three moments of time previously mentioned. This will enable understanding of how their expectations and experiences change *through time* as the intervention is being implemented.

##### PHC users—interviews of the process evaluation (n=12–18 interviewees/arm): process evaluation

For the interviews, we will include PHC users with and without chronic conditions at pre-intervention, during intervention and post-intervention. These participants do not need to be the same each time. We will consider diversity of age groups to see how perception is affected by this characteristic.

##### Healthcare workers (n=6–10 healthcare workers/arm): qualitative and process evaluation

The number of healthcare workers to interview would depend on the workforce of each PHC centre. Ideally, six healthcare workers per arm across the three arms will be interviewed, for a total of 18–22 expected interviews per country.

However, during the implementation of the capacity building component, all the healthcare workers who participated will be included in the measurement of the indicators.

### Data collection procedures

#### Quantitative evaluation

Pre-intervention, an initial survey will be conducted to estimate the prevalence of chronic conditions of interest and to validate the tools used to define the primary outcomes (online supplemental tables 2, 3). Due to the context with low awareness of HTP and type 2 diabetes, this survey will identify adults with these conditions, invite them to the study, obtain informed consent and track them every 3 months. Information will be collected using appropriate technology strategies such as REDCap templates with trained fieldworkers. The survey instrument is detailed in online supplemental material 4.

Questionnaires of the initial survey will contain primary outcomes (eg, PSQ-18, health responsiveness) as well as other indicators of interest (eg, People’s Voice Survey). Overall information will be collected as part of the initial survey, but also in five different moments during the project. As tools used to inform primary outcomes were developed in English, data collected from the initial survey will be used to validate and adapt such tools. The characteristics and timing of the outcomes are detailed in table 3. Thus, we estimate that for this survey, we will need a minimum of 420 participants per country (70 per PHC centre), stratified by sex (ie, 210 males and 210 females). In addition, some anthropometric markers such as height and weight will be measured using a stadiometer and validated scales, respectively. Blood pressure will also be measured using automated devices (OMRON HEM-780), validated for population-based studies, and using standardised procedures.^42^ Blood pressure will be evaluated after 5 min of resting and in the right arm. Measurements will be done in triplicate, with a separation of at least 1 min between measures, and the average of the second and third assessment will be used to determine blood pressure level, HTP and control.

Information from the initial survey will be used to decide the inclusion of relevant questions and topics used to assess the intervention. Primary and secondary outcomes will be evaluated at different moments (see details in table 3). Multiple assessments will be done in specific subpopulations (ie, those having a diagnosis of HTP, those having a diagnosis of type 2 diabetes or users of PHC centres). To evaluate HTP control, we plan to measure blood pressure levels according to the aforementioned techniques. For those with diabetes, random glucose sampling using a validated glucometer as well as the time of the last meal (ie, number of hours lapsed from last meal) will be considered to identify those under appropriate control as in a previous study in resource-constrained settings.^43^

Regarding the communication strategy, it will be integrated within the quantitative evaluations and process evaluation. The knowledge and habit questionnaires will contain the related outcomes (ie, understanding of the concept, risks and symptoms, prevention and control, and behaviour, intention and attitude). Outcomes of the communication strategy will be evaluated at pre and post (table 3). Assessments will also be conducted in specific populations (ie, those having HTP, diabetes and users of PHC centres).

#### Qualitative evaluation

##### Qualitative longitudinal study with health service users

Data will be collected through in-depth interviews and, when possible, if participants accept photo-elicitation, it will be used to evoke conversations during the interviews. Each interview will be conducted by two interviewers and could last 1–1.5 hours. The interview guide will explore how participants’ individual expectations and experiences using the local health services change *through time* as a result of the intervention and how this is reflected in their use, perceived value and satisfaction of the health services. Each participant will be interviewed three times. Additionally, at the end of the first and second interviews, participants will be offered the possibility of taking pictures using a disposable camera provided by the project. These pictures should illustrate what it means for participants to live with a chronic condition, including their experience using the health services. When applicable, the pictures will be used to initiate conversations in the second and third interviews. Because this would be the first time that photo-elicitation is used in the study settings, the potential response by participants is unknown. Depending on the participant’s acceptance to photo-elicitation in the first data collection moment, the research team will assess whether to continue or suspend the activity for the second moment. If participants agree, a local photo exhibition will be organised after the implementation of the interventions and all the evaluations are concluded to display a selection of the pictures. The research team will register their experiences in an individual reflexive diary, and structured summaries of debriefing meetings will be prepared for each data collection moment.

##### Qualitative study with healthcare workers

Data will be collected through interviews at three points. The qualitative study will examine the perception of healthcare workers of their roles, responsibilities and capacity to deliver services. The qualitative research team will conduct the interviews and will use the interview transcripts for data analysis.

### Economic evaluation

Data will be collected at different points in time. Facility-level data will be collected at the initial survey and at moment 5. Data on household out-of-pocket payments will be collected in parallel with the quantitative evaluation. For the ‘care as usual’ arm, guidelines and protocols for the first level of care for diabetes, HTP and NTDs will be sought to identify the procedures and supplies to provide care at each country, complemented with interviews with key informants. For the intervention arm, we will meet with the research team who participated in co-creation and co-design processes to identify activities and information details for each country. The COHESION co-creation manual will be used to list the expected activities.^44^ An interview guide with a pre-filled checklist of activities will be applied, and administrative records will be reviewed to reduce recall bias and capture resource use to implement the intervention. It is important to mention that the COVID-19 pandemic and funding issues caused a 4-year delay between co-creation and co-design, impacting costs. For that reason, we will compare real and hypothetical costs where co-design followed immediately after co-creation. Interviews to account for the resources used will be made. Healthcare workers in each PHC centre will record all inputs used in a logbook, and this information will pre-fill cost data collection forms. In addition to the healthcare perspective, we will apply a survey to capture out-of-pocket payments from households and other household costs to be included in the societal perspective.

#### Cost calculation

Cost calculation will follow a bottom-up strategy using pre-filled data collection forms to gather information on resources used, frequencies, prices and costs. Data will be collected through interviews with key informants, on-site observation and document review. Household cost assessment will involve interviews with community members to document travel time, medicines expenses, laboratory test, consultations and other inputs. We will ask if another family or community member had to accompany them and absenteeism from work.

A form will be prepared to collect data on health resources at the facility level such as services provided, number of monthly users, infrastructure utilisation, equipment and number of human resources. An employee list will identify relevant healthcare workers for the health services under study, along with a list of expected supplies and medicines according to national guidelines. We will adapt data collection forms from the WHO Mother Baby Package Costing Tool. To collect data on resources at the household level, we will interview individuals with one or more health conditions identified in the quantitative evaluation, both users and non-users of PHC services, to capture household characteristics and healthcare expenditures, including transportation costs and lost wages. Instruments will be developed using the Living Standards Measurement Study guidelines.

Costs will be classified by input, specifically into capital and recurrent cost categories. Capital costs include large equipment, buildings, one-time training for healthcare workers and social mobilisation. Recurrent costs include supplies such as drugs, syringes, consumables and small equipment; periodic training costs such as short courses; personnel costs; operation and maintenance of buildings and vehicles; and social mobilisation costs.

### Process evaluation

Quantitative data will be collected through surveys and checklists, and qualitative data through semi-structured interviews. The data on the health system indicators will be collected in both intervention and usual care arms.

For PHC users, a guide will be implemented to document (1) services users’ perceptions of the intervention by healthcare workers; (2) perceptions about changes in the treatment received at PHC; (3) perceptions about the communication through different communications channels (eg, community-based loudspeakers, radio, community word of mouth) and (4) reach of the communication strategy and their impact. Non-intervention community members will be interviewed similarly, with questions adapted to reflect their exposure to health-related radio programmes or podcasts and the messages they recall. For non-PHC service users, information will be collected to understand why they are not using the health services from the PHC. For healthcare workers, a guide will be conducted to explore (1) why healthcare workers did use or not use the training received during the intervention, (2) how the co-designed intervention was implemented in their everyday practice and (3) what is the impact of the intervention in terms of communication between healthcare workers and service users. Some questions of the qualitative evaluation study will be integrated as mentioned previously. Finally, for decision-makers, relevant public health stakeholders (regional health directorate) will be interviewed to explore the possibilities to uptake the intervention and scaling up.

As part of the communication strategy, qualitative data will be gathered through in-depth interviews conducted within the process evaluation. Information will be collected to document the comprehension of chronic diseases, the exposition and remembrance of themes and messages of spots, interview programmes or podcast. Outcomes will be evaluated once during the project intervention (table 3). Assessments will be performed within users diagnosed with HTP and diabetes.

### Analysis

#### Primary endpoint analysis

STATA 16.0 for Windows (StataCorp, College Station, Texas) will be used for statistical analysis. We will describe variables of interest, especially numerical outcomes such as patient satisfaction or health system responsiveness, using appropriate central tendency (mean, median, etc) and dispersion measures (SD, IQR, among others). An intent-to-treat analysis as well as a per-protocol analysis plan will be developed before starting statistical analysis. Given the nature of the intervention, a pre–post study with repeated measures, appropriate longitudinal and panel data analysis techniques will be used for the assessment of the outcomes. An analysis at the individual level using generalised estimating equations or linear mixed models would be preferable to flexible handling of normal or non-normal variables. These models tend to be more robust to misspecification of the variance structure since ‘sandwich’ type variance estimates are used.^45 46^ A string of consecutive observations equally spaced in time is planned to have multiple assessments pre-intervention and post-intervention. In this way, we will be able to account for two of the most common issues in this type of quasi-experimental studies: confounding and regression to the mean.^47^ Due to the lack of randomisation in our sample, other potential confounders at the individual, but also at the setting level, will be also considered.

#### Secondary endpoint analysis

Similar to the aforementioned analysis, secondary endpoint outcomes will be assessed graphically and using tabulations (eg, access to HTP care, access to diabetes care). The effect of the intervention on our outcomes will be conducted using generalised estimating equations or mixed models (linear or logistic depending on the type of variable). Potential confounders will also be considered. As usual, other analyses will be conducted to explain the effect of the intervention, if any, on main and secondary outcomes. This includes, but is not limited to, stratification by sex, analysis per specific chronic condition (eg, HTP, diabetes, country-specific NTD), etc.

#### Qualitative analysis

This study is grounded within interpretative approaches, in which interview data are seen to provide access to accounts of how respondents perceive, understand and talk about the world.^48 49^ The interviews will be recorded and transcribed verbatim in the local languages. The resulting transcripts will be analysed following the same procedure in the three countries, deductively (ie, coding data according to key elements of existing conceptual frameworks) and inductively (ie, using thematic analysis to elicit new themes or unexpected findings through coding and categorising of data) using the qualitative ATLAS.ti software. The data analysis will be done by a team speaking the local language, so no translation of the transcripts will be needed. However, to enable cross-country comparisons and the production of research outputs, synthesis tables will be completed in English by the local research teams to inform these comparison processes. Overall, intersectional-gender and sociocultural dimensions will be considered when analysing the data.

#### Economic analysis

A detailed micro-costing of the resources will be implemented, and descriptive statistics of the implementation resources and costs of the different strategies and usual care will be presented. Implementation and healthcare costs will be included in the cost-effectiveness analysis from the healthcare perspective. In addition to these costs, other direct and indirect costs will be included in the analysis from a societal perspective. The primary outcome of the economic evaluation will be the incremental cost-effectiveness ratio.

A sensitivity analysis will be performed by varying parameters such as the discount rate, among others, to account for uncertainty.

For cost-effectiveness analysis, primary outcomes indicators will be used (table 3). Assessment will consider patient satisfaction (PSQ-18) and health system responsiveness as primary outcomes. Each country team will set cut-off points to identify community satisfaction with services. Incremental cost-effectiveness ratios will be calculated as the difference in costs and effectiveness between intervention and usual care arms using both alternatives. For cost–utility analysis, we will evaluate two alternatives using EQ-5D-5L as outcome for quality-of-life measurement. Utility values will be derived by combining EQ-5D-5L results with pre-existing set of values (‘tariffs’). EQ-5D-5L and corresponding country value sets are validated in Nepal and Peru; Mozambique will use the international standard value set. Quality-adjusted life-years (QALYs) will be calculated by multiplying utility value for each alternative during 10-year incremental cost–utility ratios, which will be calculated using costs and QALY differential.

#### Process evaluation analysis

Mixed-method analysis will be conducted where quantitative and qualitative data will be systematically integrated to have results of each ‘case’. Quantitative and qualitative data will be disaggregated by sex, and interpreted and reported based on gender, acknowledging, when applicable, other intersecting social inequalities (eg, age, socioeconomic status).^50^ Quantitative data will be reported by means or SD, and in the case of surveys with categorical responses, frequencies and proportions will be estimated. Interview (qualitative) data will follow the same analysis as the qualitative studies outlined above. The information from the three countries will be contrasted.

## Ethics and dissemination

This research protocol, data collection instruments and informed consents will be reviewed and approved by the relevant Institutional Review Boards (IRB) or Ethics Committees of Universidad Peruana Cayetano Heredia (Peru), Universidade Eduardo Mondale (Mozambique) and Nepal Health Research Council (Nepal). In addition, when needed, applications to other ethical bodies will be carried out to respond to country-specific requirements. After receiving feedback from all the IRBs/ Ethics Committees, modifications will be made to the protocol. During the implementation of the study, the ethical principles outlined in the Declaration of Helsinki will be respected, and the recommendations made by the IRB will be strictly followed.

In addition, all participants will be assigned a unique identification code. Data will be stored in paper (informed consents) and electronic forms. Electronic data will be archived, copied and secured with passwords. Paper formats will be stored with access limited to specific individuals from the COHESION-I team. Personal information, including the participant’s name, age and other potential identifiers, will be stored in folders separate from the transcript database, protected by passwords. Only the COHESION-I team will have access to this information.

### Patient and public involvement

The design of the protocol draws on the initial phase of the COHESION project.^51^ In that phase, the proposed interventions were developed using a co-created methodology^37^ that incorporated perspectives from local sites in the participating countries, along with input from multilevel stakeholders, including decision-makers. The COHESION-I study has included a co-designed process of the proposed intervention employing participatory methodologies. The involvement and contributions of multiple stakeholders help ensure that the complex intervention is responsive to their needs and perspectives, with the goal of optimising research outcomes.

### Project status

The COHESION-I project began its research activities in the three countries with the co-design process. Later, the three countries started the initial survey and the following evaluations (moments). In Peru, 1,803 participants were recruited for the census in the participating sites; in Mozambique, 408; and in Nepal, 409. As of December 2025, Peru is at Moment 3, whereas Mozambique is starting Moment 2, and Nepal is at Moment 2 of the data collection.

## Discussion

To improve the management of chronic diseases in LMICs, the PHC level needs to be strengthened and prepared to provide high-quality care. The COHESION-I project will implement a complex co-created/co-designed intervention in study arms of three LMICs. Also, the project will assess the relevance of participatory processes in identifying and addressing the needs of health systems to improve patient satisfaction and responsiveness in people with chronic conditions in three different countries. Additionally, four types of evaluations will be conducted, including quantitative, qualitative, process and economic evaluation. The COHESION-I project will provide a description of the three countries’ processes; following similar procedures in diverse settings at the same time is useful in terms of cross-country learning.

Chronic diseases in LMIC also need complex interventions to find solutions to the poor management that patients are receiving. Complex interventions are characterised by multiple components that interact between them,^52^ and it is proposed that the management of chronic diseases is complex if we consider that multiple levels of care as well as the long-term needs and involvement of multiple stakeholders should be part of the pathway to identify solutions.^53^ These types of intervention can solve global health problems and can be used to strengthen the PHC level and improve the quality of health services.^54^ However, the designs of complex interventions have been historically conducted by researchers and nowadays are growing the interests of involving stakeholders in the design of complex interventions to be context-adapted.

The co-creation approach ensures the involvement of diverse stakeholders to develop an intervention and/or implement it.^15 55^ In this project, we are using co-creation and co-design; the first one refers to the development and design of an intervention with the participation of stakeholders, whereas co-design is the refinement of the components of the intervention to develop the outputs and have them ready for being delivered.^15 56^ Nevertheless, there is a lack of studies that scientifically evaluated the impact of co-created/co-designed interventions, and for that reason, the current study is relevant. It is comparing two arms against the usual care: one arm where the sites participate in co-creation and co-design and a second arm where only co-design was applied. The assumption is that co-creation is necessary to design tailored interventions, but once co-created, this intervention can be implemented in sites with similar characteristics using only co-designed.^57 58^

This study also highlighted the need to understand better the elements that contribute to patient satisfaction in low-resource settings and pay attention to the dynamics between healthcare providers and users at the PHC to ensure they are patient-centred, culturally competent and community orientated.^59^,^63^ Patient satisfaction and responsiveness are proxies of the health system performance having the point of view of the users. Nowadays, patient satisfaction has gained more attention since Kruk *et al*^9^ highlighted that high-quality health systems need to expand the focus from disease management to go beyond and place people in the centre of the complex health system. One domain in the high-quality health systems framework encompasses respect (dignity, privacy, clear communication, etc) and user focus (choice of providers, short wait times, etc). Also, in chronic care, the interaction between healthcare workers and the service users and the impact of this interaction on the user’s health is complex. For this reason, the health systems responsiveness framework works to align expectations of patients and healthcare workers having as core components dignity, autonomy, confidentiality, clear communication, prompt attention, choice of provider, quality of amenities and access to social support networks.^11^ The evaluation of patient satisfaction using the PSQ-18 and responsiveness from an individual level using the WHO’s questionnaire will provide an overview of the quality of the health system.

Another aspect of the value of COHESION-I is the comprehensive evaluation that includes quantitative measurements for the primary and secondary outcomes but also a longitudinal qualitative evaluation as well as process and an economic evaluation.^52^ It is relevant for the evaluation of interventions to not focus only on the improvement of patient satisfaction and responsiveness or in some health indicators, but also understand the rationale behind the perceptions of participants and healthcare workers about these outcomes. Also, in this study, a longitudinal approach is used to explore the experiences of participants to understand how they unfold over an extended period of time.^64^ Additionally, the process evaluation will be useful to understand why the intervention works or not; interviewing different stakeholders and including measurements of context, mechanism of impact and implementation outcomes.^32 33^ Whereas the economic evaluation will provide the perspective of the cost, cost benefit and utility of co-creation and co-design for decision-makers, but also data on out-of-pocket expenses at the individual level.^65^

This study has some limitations; there are some uncertainties about the number of participants per arm, and the initial survey will provide information about the number of cases and the current satisfaction and responsiveness of the studied population. This will allow researchers to review the sample size and make some adjustments. The intervention will be implemented in PHC centres from low-resource settings. So, this confronts us with two scenarios: on one side, the components of the intervention may not be sufficient to fulfil the needs of the population (eg, they may want medicines or more healthcare workers) due to the precarious conditions of their health system; on the other side, because these are underserved populations, it is possible that the intervention could have a major impact because they are not used to receiving interventions or health programmes.

## Conclusions

This study has NCDs and NTDs as tracer conditions to improve the health system at the PHC level in three countries. It is innovative because it focuses not on the supply side but on the demand side and uses co-creation and co-design with stakeholders from low-income settings, including rural populations. Also, it proposes a scientific evaluation of a complex co-created/co-designed intervention in three different countries using wide diverse outcomes pointing out different aspects that should be evaluated when a complex intervention is implemented.

## Supplementary material

10.1136/bmjopen-2025-109433 online supplemental file 1

## Data Availability

Data are available upon reasonable request.
