# Correction: ‘Implementation of the community health system innovation project in three low- and middle-income countries: COHESION-I study protocol’

**DOI:** 10.1136/bmjopen-2025-109433corr1

**Published:** 2026-02-18

 Lazo-Porras M, Bernabe-Ortiz A, Damasceno A, *et al.* Implementation of the community health system innovation project in three low- and middle-income countries: COHESION-I study protocol. *BMJ Open* 2025;15:e109433. doi:10.1136/bmjopen-2025–1 09 433

This article has been corrected since it was published online. The funding section in Footnotes has been updated from “COHESION-I receives funding from the National Institute for Health and Care Research under the NIHR Global Health Policy and Systems Research Programme NIHR150261” to “This research was funded by the NIHR (project NIHR150261) using UK international development funding from the UK Government to support global health research. The views expressed in this publication are those of the author(s) and not necessarily those of the NIHR or the UK government.”

