# Supplementary material for: Implementation of the community health system innovation project in three low- and middle-income countries: COHESION-I study protocol
Source: BMJ Open. 2025 Dec 31;15(12):e109433. doi: 10.1136/bmjopen-2025-109433 (PMC12911784; doi:10.1136/bmjopen-2025-109433)
Supplement: online supplemental file 1 [file bmjopen-15-12-s001.docx]

**Content**

[Supplementary Material S1. Co-created interventions 2](#_Toc213861812)

[Supplementary Material S2. Details of outcomes 3](#_Toc213861813)

[Supplementary Material S3. Details of Implementation outcomes 8](#_Toc213861814)

[Supplementary Material 4. Tools of data collection 12](#_Toc213861815)

# Supplementary Material S1. Co-created interventions

| **Component** | **Mozambique** | **Nepal** | **Peru** |
| --- | --- | --- | --- |
| Community | - Radio programs, spots, or podcasts and pamphlets informing population on hypertension, PHC and appointment system. | - Radio programs, spots, or podcasts and pamphlets to promote PHC and improve understanding of chronic conditions and timely management. - Involvement of Female Community Health Volunteers. | - Radio programs, spots, or podcasts to motivate service users and community members to clarify any concerns they may have about their health condition and treatment. |
| Healthcare workers | - Capacity building on hypertension and clear communication. - Facility based guidelines/ algorithm. - Establishment of an “information booth”. - Group discussions on challenges and opportunities. | - Capacity building on diabetes, hypertension, clear communication and prompt attention including role playing. - Flip chart and guidelines for management of diabetes and hypertension. | - Capacity building on management of diabetes, hypertension and neurocysticercosis, communication and dignity using role playing. - “Communication jar” to prompt communication between healthcare workers and service users. |
| Health facility | - Appointment system. - Advocacy on the issue of access to medicines. | - Decentralised decision making on improving facility. - Posters about diabetes, hypertension and with questions to prompt communication between healthcare workers and service users. | - Decentralised decision making on improving facility. |

# Supplementary Material S2. Details of outcomes

| **Outcome** | **Data source** | **Collected from** | **Time** |
| --- | --- | --- | --- |
| **Quantitative evaluation** | | | |
| *Primary outcomes* | | | |
| Patient satisfaction | PSQ-18^a^ | PHC users with chronic conditions (HTP^1^, DB^2^, NTD^3^)  PHC user^4^ | Initial survey  Moment 1 - 5 |
| Health system responsiveness | WHO responsiveness tool | PHC user with chronic conditions (HTP, DB, NTD)  PHC user | Initial survey  Moment 2, 3 and 4 |
| People’s Voice Survey | People’s Voice Survey | PHC user with chronic conditions (HTP, DB, NTD)  PHC user | Initial survey |
| Quality of Life | EQ-5D-5L^b^ | PHC user with chronic conditions (HTP, DB, NTD)  PHC user | Initial survey  Moment 2, 3 and 4 |
| *Secondary outcomes* | | | |
| Access to hypertension care (anti-hypertensive drugs) | Self-reported | PHC user with HT | Initial survey  Moment 2, 3 and 4 |
| Access to treatment and medication for hypertension | Self-reported | PHC user with HT | Initial survey  Moment 2, 3 and 4 |
| Level of hypertension control (blood pressure control) | Blood pressure measurement | PHC user with HT | Initial survey  Moment 2, 3 and 4 |
| Access to diabetes care (diabetic drugs) | Self-reported | PHC user with DB | Initial survey  Moment 2, 3 and 4 |
| Access to treatment and medication for diabetes | Self-reported | PHC user with DB | Initial survey  Moment 2, 3 and 4 |
| Level of diabetes control (glycemic control) | Random glucose using glucometer and strips | PHC user with DB | Initial survey  Moment 2, 3 and 4 |
| Knowledge about chronic conditions | Self-reported | PHC users with chronic conditions (HTP, DB, NTD)  PHC user | Moment 2 and 5 |
| Behaviours about salt consumption, consumption of fruits and vegetables and health service use | Self-reported | PHC user with chronic conditions (HTP, DB, NTD)  PHC user | Moment 2 and 5 |
| **Qualitative evaluation** | | | |
| Perceived value of health services | In-depth interviews | PHC users with chronic conditions (HTN, T2D, NTD) | Initial survey or moment 1, 3 and 5 |
| Patient satisfaction | In-depth interviews | PHC users with chronic conditions (HTN, T2D, NTD) | Initial survey or moment 1, 3 and 5 |
| Perceived roles and responsibilities | Interviews | HCWs^5^ | Initial survey or moment 1, 3 and 5 |
| Perceived capacity | Interviews | HCWs | Initial survey or moment 1, 3 and 5 |
| **Economic evaluation** | | | |
| Cost per satisfied patient | Facility level cost questionnaire | PHC users with chronic conditions (HTP, DB, NTD)  PHC user | Moment 5 |
| Costs of services provided | Facility level cost questionnaire | Health care providers | Moment 1 and 5 |
| Cost per patient with a positive perception of health system’s responsiveness | Facility level cost questionnaire | PHC users with chronic conditions (HTP, DB, NTD)  PHC user | Moment 5 |
| Out-of-pocket expenses | Household out-of-pocket questionnaire | PHC users with chronic conditions (HTP, DB, NTD)  PHC user | Moment 1 and 5 |
| Cost per QALY gained | Costs calculated previously | PHC users with chronic conditions (HTP, DB, NTD)  PHC user | Moment 5 |
| **Process evaluation** | | | |
| *Context* | | | |
| *Barriers and facilitators* | | | |
| Environmental context during the implementation of COHESION-I (six-months) | Field notes | Field workers | Moment 1 and 5 |
| Influences of factors on intervention goals (economic, political, social) | Field notes | Field workers | Moment 1 and 5 |
| Support of the Regional Health Directorate | Meso level questionnaire main survey | Regional decision makers | Moment 5 |
| Support of the municipality/community to the facility | Q7_Questionnaire Local leaders | Local authorities | Moment 5 |
| Support of community members and organizations to the facility | Q4_Questionnaire Patient | Community members  PHC users with chronic conditions | Moment 1 and 5 |
| Team dynamics within the health center’s organization | Team Climate Inventory (TCI) | HCWs | Moment 3 |
| *Health system-level indicators* | | | |
| Healthcare workers | (semi-structured interview)  Evaluation from the formative work:  Training received on NCDs,  Knowledge of NCDs,  Practice about NCDs, and  Self-perception of HCWs in the management of NCDs and NTDs. | HCWs | Moment 1 and 5 |
| Service Delivery in prevention and management | Evaluation of NCDs services (promotion, prevention, curative, palliative, and rehabilitation) | HCWs  PHC users with chronic conditions  Community members | Moment 1 and 5 |
| Accessibility and affordability of medicines and care | Evaluation from the formative work:  Access to diagnostic tools  Means of diagnosing and testing for the NCD  Quantification and distribution of medicines  Financial impediments and consequences | HCWs  PHC users with chronic conditions  Community members | Moment 1 and 5 |
| Patient education, self-management and empowerment | Assessment of the information and education provided to service users (content and method of delivery) | HCWs  PHC users with chronic conditions  Community members | Moment 1 and 5 |
| Community involvement | Evaluation of community organization to support and facilitate PHC services for NCDs and NTDs | HCWs | Moment 1 and 5 |
| *Implementation outcomes* | | | |
| Supplementary Material 3^2^ |  |  |  |
| *Mechanism of Impact* | | | |
| Coherence | Semi-structured interview oriented around NPT constructs | PHC user  Community members  HCWs  Regional Decision makers (only moment 5) | Moment 1, 4 (PHC user, community members)  Moment 1, 4 (HCWs)  Moment 5 |
| Cognitive participation | Semi-structured interview oriented around NPT constructs | PHC user  Community members  HCWs (No moment 3)  Decision makers (only moment 5) | Moment 2, 3, 4 (PHC user, community members)  Moment 2, 4 (HCWs)  Moment 5 |
| Collective action | Semi-structured interview oriented around NPT constructs | PHC user  Community members  HCWs (No moment 2, 3)  Decision makers (only moment 5) | Moment 2, 3, 4 (PHC user, community members)  Moment 4 (HCWs)  Moment 5 |
| Reflexing monitoring | Semi-structured interview oriented around NPT constructs | PHC user  Community members  HCWs  Local authorities (only moment 5)  Decision makers (only moment 5) | Moment 4 (PHC user, community members)  Moment 4 (HCWs)  Moment 5 |
| **Communication strategy^3^** |  |  |  |
| *From quantitative evaluation* |  |  |  |
| Comprehension of the concept of chronic disease | Questionnaire of knowledge | PHC user  PHC user with chronic conditions (HTP and DB) | Moment 1 and 5 |
| Knowledge about risks, symptoms, prevention/ control | Questionnaire of knowledge | PHC user  PHC user with chronic conditions (HTP and DB) | Moment 1 and 5 |
| Present behaviour, intention and attitude (consumption of salt, fruits and vegetable, and health monitoring attendance) | Questionnaire on lifestyle habits | PHC user  PHC user with chronic conditions (HTP and DB) | Moment 1 and 5 |
| *From process evaluation* |  |  |  |
| Exposure of themes and/or messages from spots, interview programmes or radio soap operas | Semi-structured interviews | PHC user with chronic conditions (HTP and DB) | Moment 3 |
| Remembrance of themes and/or messages from spots, interview programmes or radio soap operas | Semi-structured interviews | PHC user with chronic conditions (HTP and DB) | Moment 3 |
| Comprehension of chronic diseases | Semi-structured interviews | PHC user with chronic conditions (HTP and DB) | Moment 3 |

^1^ HTP: Hypertension ^2^ DB: Diabetes ^3^ NTD: Neglected Tropical Diseases ^4^ PHC user: User who received primary health care in one of the health facilities ^5^ HCWs: Healthcare workers

^a^ PSQ-18: Patient Satisfaction Questionnaire Short Form ^b^ EQ-5D-5L: EuroQol 5 dimensions 5 levels

^2^ For the implementation outcome from the Process Evaluation, the Supplemental Material 3 are developed below

^3^ The evaluation of the communication strategy will be integrated into the quantitative and process evaluation

# Supplementary Material S3. Details of Implementation outcomes

| Activity | Outcome or indicator | Data source | Collected from | Countries | Time |
| --- | --- | --- | --- | --- | --- |
| Co-design | | | | | |
| Co-design workshops | Reach | Semi-structured interview   - Communication strategies | PHC user  Community members | All | Moment 1 |
|  | Acceptability | Semi-structured interview   - Intervention components | HCWs  PHC user  Community members | All | Moment 1 |
|  | Feedback | Semi-structured interview   - Workshops dynamics | HCWs  PHC user  Community members | All | Moment 1 |
| Community Component |  |  |  |  |  |
| Radio programs, spots or podcast | Reach | Semi-structured interview | PHC user  Community members | All | Moment 2, 3, 4 and 5 |
|  | Reach and remembrance | Survey | PHC user with chronic conditions | All | Moment 5 |
|  | Dose | Semi-structured interview | PHC user  Community members | All | Moment 3, 4 and 5 |
|  | Acceptability | Semi-structured interview | PHC user  Community members | All | Moment 4 |
|  | Feedback | Semi-structured interview | PHC user  Community members | All | Moment 4 |
| Communication Jar / System card | Acceptability | Semi-structured interview | PHC user  Community members | Peru | Moment 4 |
|  | Feedback | Semi-structured interview | PHC user  Community members | Peru | Moment 4 |
| Leaflets/ Pamphlets | Reach | Semi-structured interview | PHC user  Community members | Mozambique  Nepal | Moment 2, 3, 4  Moment 5 |
|  | Acceptability | Semi-structured interview | PHC user  Community members | Mozambique  Nepal | Moment 4 |
| FCHVs mobilization* | Reach | Semi-structured interview | PHC user  Community members | Nepal  Mozambique | Moment 2, 3, 4 and 5 |
|  | Acceptability | Semi-structured interviews | PHC user  Community members | Nepal  Mozambique | Moment 4 |
| Healthcare workers component | | | | | |
| Capacity building for HCWs | Dose | Field notes | HCWs | All | Moment 3 |
|  | Reach | Field notes  Intervention records | HCWs | All | Moment 3 |
|  | Fidelity (quality) | Semi-structured interview | HCWs | All | Moment 3 |
|  | Fidelity (training) | Field notes  Fidelity checklist | HCWs | All | Moment 3 |
|  | Acceptability | Self-complete questionnaires Kirkpatrick’s (only moment 3) | HCWs | All | Moment 3 |
|  | Knowledge | Checklist | HCWs | All | Moment 3 |
|  | Adaptation | Semi-structured interview | HCWs and FCHV | All | Moment 4 |
| Capacity building for FCHV | Fidelity (training) | Field notes  Fidelity checklist | FCHV  Health Committees members | Nepal  Mozambique | Moment 3 |
|  | Fidelity (quality) | Semi-structured interview | FCHV  Health Committees members | Nepal  Mozambique | Moment 3 |
|  | Dose | Field notes | FCHV  Health Committees members | Mozambique | Moment 3 |
|  | Reach | Field notes  Intervention records | FCHV  Health Committees members | Nepal  Mozambique | Moment 3 |
|  | Acceptability | Self-complete questionnaires Kirkpatrick’s (only moment 3) | FCHV  Health Committees members | Nepal  Mozambique | Moment 3 |
|  | Knowledge | Checklist during the training  Knowledge test for each module | FCHV  Health Committees members | Nepal  Mozambique | Moment 3 |
| Communication Jar / System card | Acceptability | Semi-structured interview | HCWs | Peru | Moment 4 |
|  | Feedback | Semi-structured interview | HCWs | Peru | Moment 4 |
| Flipcharts, algorithm and/or guidelines | Acceptability | Semi-structured interview | HCWs | All | Moment 4 |
|  | Feedback | Semi-structured interview | HCWs | All | Moment 4 |
|  | Fidelity (training) | Field notes  Fidelity checklist | HCWs | All | Moment 3 |
| Information booth | Fidelity | Field notes  Observations | HCWs  Local authorities  Community members | Mozambique | Moment 3 |
|  | Reach | Field notes and records of the number of people that receive care a certain week | HCWs  Local authorities  Community members | Mozambique | Moment 4 |
|  | Acceptability | Interviews | HCWs  Local authorities  Community members | Mozambique | Moment 4 |
| Health facility component | | | | | |
| Advocacy on access to medicines | Fidelity | Field notes | HCWs  Local authorities  Community members | Mozambique | Moment 4 |
| Facility grant | Acceptability | Semi-structured interview | HCWs  Local authorities  Community members | Nepal  Peru | Moment 4 |
| Poster | Acceptability | Semi-structured interview | HCWs  Local authorities  Community members | Nepal | Moment 4 |

* Only for Mozambique

# Supplementary Material 4. Tools of data collection

**I. SOCIO-DEMOGRAPHIC QUESTIONS**

Module: Survey Data, Location, and Date of Survey

1. Interviewer's initials: _________________________

2. Interviewer code: __________________________

3. Survey Date (Day/Month/Year): ________/___________/__________

4. Interview Time: _____:_____ AM / PM

5. Name of the place/population center where the survey is being conducted:

District: ______________________

Population center: _______________

Module: Informed Consent

Has the consent been read to the interviewee? Yes No

Has the interviewee agreed to participate in the study? Yes No

Has the respondent's written consent been obtained? Yes No

Module: participant identification data

1. Participant's ID document number: ____________________________________

2. Lab Sample Code: _________________________________________

3. Complete family names: _____________________________________________

4. First and middle names: ______________________________________________

5. Participant's address: ________________________________________________

6. Phone(s) to contact the participant:

Participant's cell phone: ___________________________

Participant's landline phone: _________________________

Telephone number of relative, friend or neighbor (for contact): ______________

Module: socio-demographic data of the interviewee

1. Sex

🔾 Male

🔾 Female

2. Age: _________ years old (as today)

3. What is your marital status?

- Single
- Cohabitant (with current partner)
- Married
- Separate
- Divorced
- Widower
- Refuse to respond

4. Please indicate the highest level of education you have completed:

- - No study in educational institutions.
  - Primary Not Completed (Interrupted)
  - Completed Primary School
  - Secondary Not Completed (Interrupted)
  - Completed Secondary School
  - Interrupted technical or military/police studies
  - Technical or military/police studies in progress or completed
  - Interrupted Undergraduate University Studies
  - Undergraduate university studies in process or completed
  - Other: _____________________________________________________________

5. Are you currently employed?

- - Yes 🡪 what do you do? _____________
  - No

Module: on media consumption (radio, social networks, etc.)

6. Do you often listen to radio (e.g. news or comedy programs on AM or FM)?

- - No 🡪 Skip to question 8
  - Yes

7. Please, mark the days of the week when you usually listen to the radio (e.g. news or comedy programs on AM or FM). You can mark many days of the week. Also, indicate the times when you usually tune in to the radio

- Monday 🡪 Between what hours, approximately? _____________
- Tuesday 🡪 Between what hours, approximately? _____________
- Wednesday 🡪 Between what hours, approximately? _____________
- Thursday 🡪 Between what hours, approximately? _____________
- Friday 🡪 Between what hours, approximately? _____________
- Saturday 🡪 Between what hours, approximately? _____________
- Sunday 🡪 Between what hours, approximately? _____________

8. Do you regularly watch videos on social networks (e.g. YouTube, TikTok, etc.)?

- No🡪 Skip to Next Questionnaire (FINDRISC)
- Yes

9. What types of videos do you usually prefer to watch?

- Direct video (e.g., broadcasts that occur as the events are happening)
- Recorded/recorded material (e.g. videos with content produced prior to broadcast)
- I have no preference; I am indifferent.

10. Now, please rank the following options from the one you use the most to watch videos (1st place), to the one you use the least (6th place)

| Social network | Ranking position | | | | | |
| --- | --- | --- | --- | --- | --- | --- |
|  | 1^st^ | 2^nd^ | 3^rd^ | 4^th^ | 5^th^ | 6^th^ |
| TikTok | 🔾 | 🔾 | 🔾 | 🔾 | 🔾 | 🔾 |
| YouTube | 🔾 | 🔾 | 🔾 | 🔾 | 🔾 | 🔾 |
| Instagram | 🔾 | 🔾 | 🔾 | 🔾 | 🔾 | 🔾 |
| Facebook | 🔾 | 🔾 | 🔾 | 🔾 | 🔾 | 🔾 |
| WhatsApp | 🔾 | 🔾 | 🔾 | 🔾 | 🔾 | 🔾 |
| Twitter | 🔾 | 🔾 | 🔾 | 🔾 | 🔾 | 🔾 |

**II. FINDRISC QUESTIONNAIRE**

1. Do you usually have at least 30 minutes of physical activity at work and/or during leisure time daily (including normal daily activity)?

- Yes
- No

2. How often do you eat vegetables, fruit, or berries?

- Every day
- Not every day

3. Have you ever taken antihypertensive medication regularly?

- No
- Yes

4. Have you ever been found to have high blood glucose (e.g., in a health examination, during an illness, during pregnancy)?

- No
- Yes

5. Have any of the members of your immediate family or other relatives been diagnosed with diabetes (type 1 or type 2)?

- No
- Yes: grandparent, aunt, uncle, or first cousin (but no own parent, brother, sister or child)
- Yes: parent, brother, sister, or own child

**III. STEPS QUESTIONNAIRE**

History of Elevated Blood Pressure

1. When was the last time your blood pressure was measured by a healthcare professional?

- In the last 12 months
- Between 1 and 5 years
- More than 5 years
- Never

2. Have you ever been told by a doctor or other health worker that you have raised blood pressure or hypertension?

- No
- Yes

3. Are you currently receiving any of the following treatments/advice for high blood pressure prescribed by a doctor or other health worker?

3.1. Special diet by medical prescription

- No
- Yes

3.2. Advice or treatment to lose weight

- No
- Yes

3.3. Advice or treatment to stop smoking

- No
- Yes

3.4. Advice to start or do more exercise

- No
- Yes

3.5. Drugs (medication) that you have taken in the past two weeks

- No
- Yes 🡪 Indicate which of the following:
- Amlodipine
- Atenolol
- Captopril
- Enalapril
- Hydrochlorothiazide
- Losartan

4. During the past 12 months, have you seen a traditional healer for raised blood pressure or hypertension?

- No
- Yes

5. Are you currently taking any herbal or traditional remedy for your raised blood pressure?

- No
- Yes

History of Diabetes

6. When was the last time your blood glucose was measured by a healthcare professional?

- In the last 12 months
- Between 1 and 5 years
- More than 5 years
- Never

7. In the past 12 months, have you had your blood sugar measured by a doctor or other health worker?

- No
- Yes

8. Have you ever been told by a doctor or other health worker that you have raised blood sugar or diabetes?

- No
- Yes

9. Are you currently receiving any of the following treatments/advice for diabetes prescribed by a doctor or other health worker?

9.1. Special prescribed diet

- No
- Yes

9.2. Advice or treatment to lose weight

- No
- Yes

9.3. Advice or treatment to stop smoking

- No
- Yes

9.4. Advice to start or do more exercise

- No
- Yes

9.5. Drugs (medication) that you have taken in the past two weeks

- No
- Yes 🡪 Indicate which of the following:
- Glibenclamide
- Human insulin (recombinant DNA)
- Insulin Human Isophane (Nph)
- Metformin

9.6. Insulin

- No
- Yes

10. During the past 12 months, have you seen a traditional healer for diabetes or

raised blood sugar?

- No
- Yes

11. Are you currently taking any herbal or traditional remedy for your diabetes?

- No
- Yes

History of Neglected Tropical Diseases (NTDs)

11. Have you ever been diagnosed with any tropical disease, such as neurocysticercosis?

(Schistosomiasis (Mozambique) / Filariasis (India) / Leprosy (Nepal))

- No 🡪 Skip to Next Questionnaire (EQ-5D-5L)
- Yes

12. Are you currently taking any medication for the tropical diseases we have just mentioned?

- No
- Yes 🡪 What medication(s)?: __________________________________________

13. Are you taking medication for any disease or health condition other than diabetes or high blood pressure?*

- No
- Yes 🡪 For which disease or health condition? __________________________
- What is the name of the medication? _________________________
- Does not know / does not remember

**IV. EQ-5D-5L QUESTIONNAIRE**

Under each heading, please tick the ONE box that best describes your health TODAY

MOBILITY

- I have no problems in walking about
- I have slight problems in walking about
- I have moderate problems in walking about
- I have severe problems in walking about
- I am unable to walk about

SELF-CARE

- I have no problems washing or dressing myself
- I have slight problems washing or dressing myself
- I have moderate problems washing or dressing myself
- I have severe problems washing or dressing myself
- I am unable to wash or dress myself

USUAL ACTIVITIES (e.g., work, study, housework, family or leisure activities)

- I have no problems doing my usual activities
- I have slight problems doing my usual activities
- I have moderate problems doing my usual activities
- I have severe problems doing my usual activities
- I am unable to do my usual activities

PAIN / DISCOMFORT

- I have no pain or discomfort
- I have slight pain or discomfort
- I have moderate pain or discomfort
- I have severe pain or discomfort
- I have extreme pain or discomfort

ANXIETY / DEPRESSION

- I am not anxious or depressed
- I am slightly anxious or depressed
- I am moderately anxious or depressed
- I am severely anxious or depressed
- I am extremely anxious or depressed

The best health imaginable

| We would like to know how good or bad your health is TODAY. |
| --- |
| The scale is numbered from 0 to 100. |
| 100 represents the best health you can imagine.  0 represents the worst health you can imagine. |
| Mark an X on the scale position that indicates how your health is TODAY. |
| Now, in the box below, write the number you marked on the scale. |

YOUR HEALTH TODAY

10

0

20

30

40

50

60

80

70

90

100

5

15

25

35

45

55

75

65

85

95

The worst health imaginable

**V. QUESTIONS RELATED TO THE USE OF THE HEALTH SYSTEM IN YOUR COMMUNITY**

Thank you for telling us, so far, about aspects related to your state of health and related treatments. Now we'd like to know a little more about your experience using the healthcare system in your community, and related topics, to better understand about it.

1. What health facility have you been attending the most times, during the last year? ________________________________________________________________________

2. Do you receive treatment for any illness or health condition (e.g., medications, nutritional or psychological counseling, etc.) at the health facility?

- - Skip 🡪 to the next questionnaire (PSQ-18)
  - Yes 🡪, which illness or condition? ______________________________________

3. How long have you been treated at this health facility?

For __________________ ( 🔾 days/ 🔾 weeks/ 🔾 months/ 🔾 years)

4. Were you previously being medically treated in another health facility?

- No
- Yes 🡪 why were you attending to other health facility? _________________________

🡪 How long ago? ______________________________________________

5. How often do you usually go to your current health facility?

Approximately, every ______________ ( 🔾 days/ 🔾 weeks/ 🔾 months/ 🔾 years)

6. Do you feel that the frequency with which you go to the health facility is sufficient to treat your illness or health condition?

- No
- Yes

7. Most of the time you go to the health facility, do you go alone or are you accompanied?

- Alone
- Accompanied 🡪 with whom?____________________________________________

8. Nowadays, how long does it take you to get to the health facility from your home? ________________________ ( 🔾 minutes / 🔾 hours)

9. Do you have current health insurance?

- No 🡪 skip to the next questionnaire (PSQ-18)
- Yes

10. What health insurance do you have? (Consider insurances available by country)

- Public health insurance 🡪 Indicate which of the following:
- Alternative 1
- Alternative 2
- Other:___________________________
- Private health insurance 🡪 Indicate which ______________________________

**VI. PSQ-18 QUESTIONNAIRE**

1. Doctors are good about explaining the reason for medical tests

- Strongly agree
- Agree
- Uncertain
- Disagree
- Strongly disagree

2. I think my doctor's office has everything needed to provide complete medical care

- Strongly agree
- Agree
- Uncertain
- Disagree
- Strongly disagree

3. The medical care I have been receiving is just about perfect

- Strongly agree
- Agree
- Uncertain
- Disagree
- Strongly disagree

4. Sometimes doctors make me wonder if their diagnosis is correct

- Strongly agree
- Agree
- Uncertain
- Disagree
- Strongly disagree

5. I feel confident that I can get the medical care I need without being set back financially

- Strongly agree
- Agree
- Uncertain
- Disagree
- Strongly disagree

6. When I go for medical care, they are careful to check everything when treating and examining me

- Strongly agree
- Agree
- Uncertain
- Disagree
- Strongly disagree

7. I have to pay for more of my medical care than I can afford

- Strongly agree
- Agree
- Uncertain
- Disagree
- Strongly disagree

8. I have easy access to the medical specialists I need

- Strongly agree
- Agree
- Uncertain
- Disagree
- Strongly disagree

9. Where I get medical care, people have to wait too long for emergency treatment

- Strongly agree
- Agree
- Uncertain
- Disagree
- Strongly disagree

10. Doctors act too businesslike and impersonal toward me

- Strongly agree
- Agree
- Uncertain
- Disagree
- Strongly disagree

11. My doctors treat me in a very friendly and courteous manner

- Strongly agree
- Agree
- Uncertain
- Disagree
- Strongly disagree

12. Those who provide my medical care sometimes hurry too much when they treat me

- Strongly agree
- Agree
- Uncertain
- Disagree
- Strongly disagree

13. Doctors sometimes ignore what I tell them

- Strongly agree
- Agree
- Uncertain
- Disagree
- Strongly disagree

14. I have some doubts about the ability of the doctors who treat me

- Strongly agree
- Agree
- Uncertain
- Disagree
- Strongly disagree

15. Doctors usually spend plenty of time with me

- Strongly agree
- Agree
- Uncertain
- Disagree
- Strongly disagree

16. I find it hard to get an appointment for medical care right away

- Strongly agree
- Agree
- Uncertain
- Disagree
- Strongly disagree

17. I am dissatisfied with some things about the medical care I receive

- Strongly agree
- Agree
- Uncertain
- Disagree
- Strongly disagree

18. I am able to get medical care whenever I need it

- Strongly agree
- Agree
- Uncertain
- Disagree
- Strongly disagree

**VII. RESPONSIVENESS QUESTIONNAIRE**

*Dimension: Dignity*

1. In the last 12 months, when you sought care, how often did doctors, nurses, or other health care providers treat you with respect?

- Always
- Usually
- Sometimes
- Never

2. In the last 12 months, how often did the office staff, such as receptionists or clerks there, treat you with respect?

- Always
- Usually
- Sometimes
- Never

3. In the last 12 months, how often were your physical examinations and treatments done in a way that your privacy was respected?

- Always
- Usually
- Sometimes
- Never

4. Overall, how would you rate your experience of getting treated with dignity at the health services in the last 12 months?

- Very good
- Good
- Moderate
- Bad
- Very bad

*Dimension: Clear communication*

1. In the last 12 months, how often did doctors, nurses, or other health care providers listen carefully to you?

- Always
- Usually
- Sometimes
- Never

2. In the last 12 months, how often did doctors, nurses, or other health care providers explain things in a way you could understand?

- Always
- Usually
- Sometimes
- Never

3. In the past 12 months, how often did doctors, nurses, or other health care providers give you time to ask questions about your health problem or treatment?

- Always
- Usually
- Sometimes
- Never

4. Overall, how would you rate your experience of how well health care providers communicated with you in the last 12 months?

- Very good
- Good
- Moderate
- Bad
- Very bad

*Dimension: Autonomy*

1. In the last 12 months, when you went for health care, were any decisions made about your care, treatment (giving you drugs, for example) or tests?

- Yes
- No

2. In the last 12 months, how often did doctors, nurses or other health care providers involve you as much as you wanted to be in deciding about the care, treatment or tests?

- Always
- Usually
- Sometimes
- Never

3. In the last 12 months, how often did doctors, nurses or other health care providers ask your permission before starting the treatment or tests?

- Always
- Usually
- Sometimes
- Never

4. Overall, how would you rate your experience of getting involved in making decisions about your care or treatment as much as you wanted in the last 12 months?

- Very good
- Good
- Moderate
- Bad
- Very bad

*Dimension: Confidentiality of information*

1. In the last 12 months, how often were talks with your doctor, nurse or other health care provider done privately, so other people who you did not want to hear could not overhear what was said

- Always
- Usually
- Sometimes
- Never

2. In the last 12 months, how often did your doctor, nurse or other health care provider keep your personal information confidential? This means that anyone whom you did not want informed could not find out about your medical conditions

- Always
- Usually
- Sometimes
- Never

3. Overall, how would you rate your experience of the way the health services kept information about you confidential in the last 12 months?

- Very good
- Good
- Moderate
- Bad
- Very bad

*Dimension: Choice of provider*

1. In the last 12 months, with the doctors, nurses and other health care providers available to you, how big a problem, if any, was it to get to a health care provider you were happy with?

- No problem
- Mild problem
- Moderate problem
- Severe problem
- Extreme Problem

2. Over the last 12 months, how big a problem, if any, was it to get to use other health care services other than the one you usually went to?

- No problem
- Mild problem
- Moderate problem
- Severe problem
- Extreme Problem

3. Overall, how would you rate your experience of being able to use a health care provider or service of your choice over the last 12 months?

- Very good
- Good
- Moderate
- Bad
- Very bad

**VIII. HEALTH SECURITY QUESTIONNAIRE**

1. Health security

1.1. How confident are you that you would receive good quality healthcare if you became very sick?

- Very confident
- Somewhat confident
- Not too confident
- Not at all confident

1.2. How confident are you that you would be able to afford the healthcare you needed if you became very sick?

- Very confident
- Somewhat confident
- Not too confident
- Not at all confident

2. Endorsement of health

2.1. Thinking about the past two years, would you say your country’s health system is getting better, staying the same, or getting worse?

- Getting better
- Staying the same
- Getting worse

2.2. Which of these statements do you agree with the most?

- Our healthcare system has so much wrong with it that we need to completely rebuild it.
- There are some good things in our healthcare system, but major changes are needed to make it work better.
- On the whole, the system works pretty well and only minor changes are necessary to make it work better.

3. Government Responsiveness to Public Input

3.1. How confident are you that the government considers the public’s opinion when making decisions about the healthcare system?

- Very confident
- Somewhat confident
- Not too confident
- Not at all confident

4. Government COVID-19 management

4.1. Overall, how would you rate the quality of the government or public healthcare system in your country during the COVID-19 pandemic?

- Excellent
- Very good
- Good
- Fair
- Poor

**IX. ASPECTS OF USER EXPERIENCE QUESTIONNAIRE**

Now, we would like to ask you a few questions to better understand your experience of being cared for at your health center. To do this, we will ask you a few brief questions.

1. The doctor or other health care professional takes enough time to tell you about the status of your illness or health condition

- Never
- Almost never
- Sometimes
- Almost always
- Always

2. Health care professionals ask you if you have any questions or doubts about your disease or your medications

- Never
- Almost never
- Sometimes
- Almost always
- Always

3. Has a health professional explained to you the possible side effects of your medication, so that you can be aware of them?

- No, they didn't explain anything or almost nothing to me about the side effects
- Yes, but the explanation was incomplete about the side effects
- Yes, the explanation was complete about the side effects

4. Medications prescribed to me are available in a timely manner at the pharmacy

- Never
- Almost never
- Sometimes
- Almost always
- Always

5. Regarding your expectations, the care you have received at the health center has been:

- Much worse than I expected
- Worse than I expected
- Same or similar to what I expected
- Better than I expected
- Much better than I expected

**X. BLOOD PRESSURE + ANTHROPOMETRIC MEASUREMENTS**

1. Blood Pressure (Arm)

|  | Measure 1 | Measure 2 | Measure 3 |
| --- | --- | --- | --- |
| Systolic pressure (arm) | _______ mm Hg | _______ mm Hg | _______ mm Hg |
| Diastolic pressure (arm) | _______ mm Hg | _______ mm Hg | _______ mm Hg |
| Pulse | _______ beats/min | _______ beats/min | _______ beats/min |
| Cuff used | _______ [1 = Small; 2 = Medium; 3 = Large] | | |
| Device Number | _______________ | | |
| Measurements made on the right side? | _______ [1 = Yes; 2 = No] | | |

2. Weight

| Weight | ______.____kg | Clothing:_____ | [1=Minimal clothing;  2=Full clothing] |
| --- | --- | --- | --- |
| Device/machine number | _______________ | | |

3. Height

| Height (standing) | _______._____ | metres |
| --- | --- | --- |

4. Abdominal circumference:

Measure 1: ______.____ cm

Measure 2: ______.____ cm

Number of centimeter used: ____
